# Exploring Epigenetic Regulation of Fear Memory and Biomarkers Associated with Post-Traumatic Stress Disorder

**DOI:** 10.3389/fpsyt.2013.00062

**Published:** 2013-07-01

**Authors:** Stephanie A. Maddox, Glenn E. Schafe, Kerry J. Ressler

**Affiliations:** ^1^Yerkes National Primate Research Center, Atlanta, GA, USA; ^2^Department of Psychiatry and Behavioral Sciences, Emory University School of Medicine, Atlanta, GA, USA; ^3^Department of Psychology, Yale University, New Haven, CT, USA; ^4^Interdepartmental Neuroscience Program, Yale University, New Haven, CT, USA; ^5^Howard Hughes Medical Institute, Chevy Chase, MD, USA

**Keywords:** amygdala, fear memory, consolidation, reconsolidation, extinction, biomarkers, PTSD

## Abstract

This review examines recent work on epigenetic mechanisms underlying animal models of fear learning as well as its translational implications in disorders of fear regulation, such as Post-traumatic Stress Disorder (PTSD). Specifically, we will examine work outlining roles of differential histone acetylation and DNA-methylation associated with consolidation, reconsolidation, and extinction in Pavlovian fear paradigms. We then focus on the numerous studies examining the epigenetic modifications of the Brain-derived neurotrophin factor (*BDNF)* pathway and the extension of these findings from animal models to recent work in human clinical populations. We will also review recently published data on FKBP5 regulation of glucocorticoid receptor function, and how this is modulated in animal models of PTSD and in human clinical populations via epigenetic mechanisms. As glucocorticoid regulation of memory consolidation is well established in fear models, we examine how these recent data contribute to our broader understanding of fear memory formation. The combined recent progress in epigenetic modulation of memory with the advances in fear neurobiology suggest that this area may be critical to progress in our understanding of fear-related disorders with implications for new approaches to treatment and prevention.

## Introduction

Pavlovian fear conditioning has become a useful tool for the identification of the cellular and molecular mechanisms necessary for the formation of fear memories (Levenson and Sweatt, 2005; Monsey et al., 2011; Mahan and Ressler, 2012; Zovkic and Sweatt, 2013; Zovkic et al., 2013). Moreover, Pavlovian fear conditioning as a model of traumatic memory formation has aided in the identification of potential intervention strategies, including extinction and reconsolidation-based memory interventions for the alleviation of traumatic fear memories (Andero and Ressler, 2012; Steckler and Risbrough, 2012). Recent work examining the cellular and molecular mechanisms necessary for the formation of Pavlovian fear memories has highlighted the significance of epigenetic mediation of traditional genomic targets already known to be critical for the formation of fear memories in animal models of post-traumatic stress disorder (PTSD). This review will address the recent progress that has been made in uncovering the epigenetic mechanisms necessary for auditory fear memory consolidation, reconsolidation, and extinction. We then address the translation of these animal findings into more recent work examining the correlation of epigenetic modifications at specific gene promoters associated with clinical PTSD.

## Epigenetic Mechanisms Required for Auditory Fear Memory Consolidation

Decades of work have established that auditory fear memory consolidation requires genomic signaling cascades to mediate the transcriptional and translational processes which ultimately underlie fear memory formation (Johansen et al., 2011). Specifically, auditory fear conditioning results in the activation of ERK/MAPK within lateral amygdala (LA) neurons (Schafe et al., 2000). ERK/MAPK in turn translocates to the nucleus where it phosphorylates the transcription factor CREB to mediate downstream transcriptional activation (Josselyn et al., 2001; Ressler et al., 2002; Ploski et al., 2010). While much progress has been made examining these traditional genomic signaling mechanisms, it has become increasingly evident that additional mechanisms likely also influence and regulate the transcriptional access necessary for synaptic plasticity and memory formation (Levenson and Sweatt, 2005, 2006; Barrett and Wood, 2008; Jiang et al., 2008; Zovkic and Sweatt, 2013; Zovkic et al., 2013). Thus in recent years attention has turned to examining how “epigenetic” mechanisms may regulate transcriptional access to genes which are critical for memory formation.

**Figure 1:**
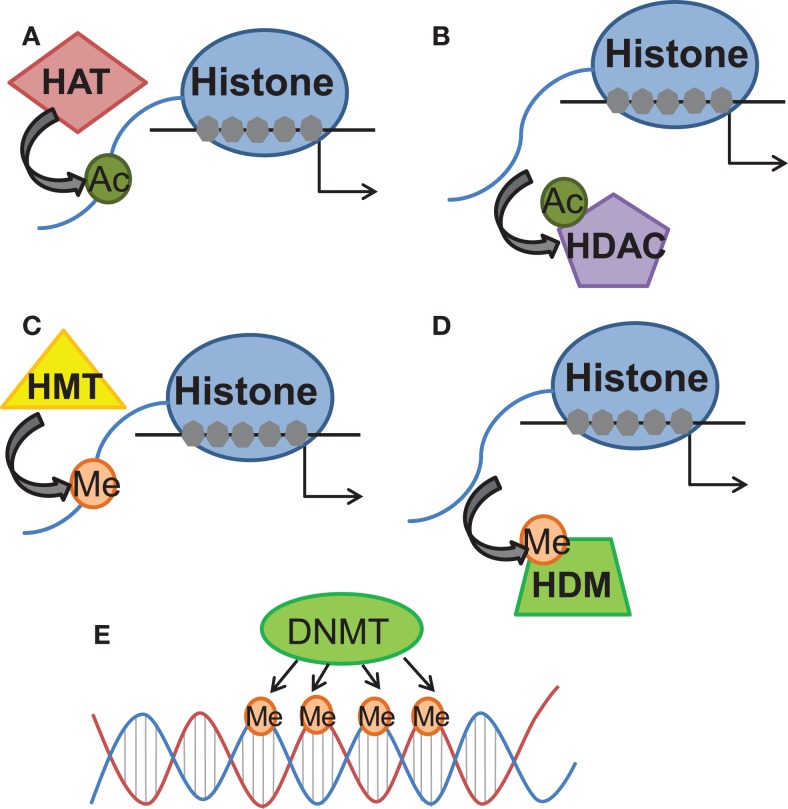
**Schematic diagram of histone and DNA-methylation processes of epigenetic regulation of gene expression**. The schematic diagram demonstrates the primary known functions of the different enzymes referred to within the review. **(A)** Histone acetyltransferases (HAT) add acetyl groups to histones, generally associated with relaxing wound DNA. **(B)** Histone deacetylases (HDAC) remove those acetyl groups. **(C)** Histone methyltransferases (HMT) add methyl groups to histones, generally associated with tightening wound DNA. **(D)** Histone demethylases (HDM) remove those methyl groups. **(E)** DNA methyltransferases (DNMT) add methyl groups to DNA, sometimes associated with DNA silencing.

Epigenetics involves the study of changes in gene expression which occur independent of alterations to the underlying DNA sequence. Two epigenetic mechanisms in particular have been examined in memory formation and synaptic plasticity: post-translational modifications to chromatin structure and DNA methylation (Levenson and Sweatt, 2005). Within the nucleus, DNA is tightly condensed into chromatin consisting of eight histones: two copies each of H2A, H2B, H3, and H4 as well as the linker histone H1. Each histone possesses an N-terminus tail capable of undergoing multiple modifications, including acetylation, phosphorylation, and methylation (Levenson and Sweatt, 2005). While a few studies have identified the regulation of histone methylation and phosphorylation accompanying fear conditioning in contextual fear paradigms (Chwang et al., 2006; Gupta et al., 2010; Gupta-Agarwal et al., 2012), histone acetylation has been more commonly studied within the context of learning and memory (Graff and Tsai, 2013). Positively charged lysine residues on N-terminus histone tails restrict transcriptional access and the acetylation of these residues via histone acetyltransferases (HATs) neutralizes the positive charges on the histone tails to relax chromatin structure and promote accessibility for transcription factor binding (Varga-Weisz and Becker, 1998; Yang and Seto, 2007). Conversely, histone acetylation is negatively regulated by histone deacetylases (HDACs) which remove acetyl groups from lysine residues and thus condense chromatin structure and interfere with transcriptional access (Varga-Weisz and Becker, 1998; Yang and Seto, 2007) (Figure 1).

Whereas histone acetylation has been widely associated with transcriptional activation, DNA methylation has traditionally been considered a static process associated with transcriptional repression (Miranda and Jones, 2007). DNA methylation is the process whereby methyl groups are added to cytosine residues within the DNA sequence and the addition of these methyl groups has been shown to inhibit transcriptional access to DNA, a process which is catalyzed by DNA methyltransferases (DNMTs) (Miranda and Jones, 2007). While the existence of dynamic DNMT activity within the mature central nervous system (CNS) has been documented, preliminary evidence has emerged to reveal a role for Gadd45b as a regulator of active DNA demethylation within the CNS (Leach et al., 2012; Sultan et al., 2012) (Figure 1).

Early evidence of a role for chromatin modifications accompanying learning in mammalian models of learning and memory was revealed by a study employing a contextual fear conditioning paradigm known to regulate the ERK/MAPK–CREB genomic signaling pathway. This study from David Sweatt’s group revealed robust regulation of histone H3 acetylation, but not H4, 60 min following contextual fear conditioning in area CA1 of the hippocampus (Levenson et al., 2004), a pattern of findings which has been well-replicated (Lubin and Sweatt, 2007; Miller et al., 2008). Further, increasing histone acetylation via HDAC inhibition has been well documented to enhance memory consolidation in a variety of hippocampal-dependent learning paradigms, including object recognition and contextual fear conditioning (Levenson et al., 2004; Stefanko et al., 2009; but see Sintoni et al., 2013).

While regulation of histone H3 and H4 acetylation have been most commonly examined and acetyl-H3 regulation is most widely reported, evidence has emerged implicating the regulation of histone H2B acetylation accompanying contextual fear and spatial learning in the hippocampus suggesting not only that there may be differential regulation of specific histones with varying types of memory formation but also that a closer look at the regulation of other histones with learning and memory is warranted (Bousiges et al., 2013). Further, it is worth noting that learning-related alterations in histone acetylation have not been ubiquitously observed. One such study employing an invertebrate model of context-signal memory, whereby presentation of a danger stimulus elicits an escape response, demonstrated that while HDAC inhibition was capable of enhancing the escape response and accompanying levels of histone acetylation with weak training procedures, only strong training was capable of resulting in robust regulation of histone acetylation (Federman et al., 2009). Further, another study employing a food aversion paradigm in the mollusk revealed asymmetric regulation of histone acetylation accompanying training (Danilova et al., 2010). These studies suggest that there may be important training-related gradients which determine the extent to which histone acetylation is regulated by learning and that there may be asymmetric differences in the engagement of histone acetylation accompanying training. However, despite these findings from invertebrate learning and memory models, the training parameters necessary to induce alterations in histone acetylation events or the existence of asymmetric regulation within mammalian models has yet to be determined.

Further examination of the regulation of histone acetylation in mammalian models has demonstrated that the observed training-related regulation of histone H3 acetylation is downstream of ERK/MAPK signaling, as the MEK inhibitor U0126 was found to impair the acetylation of H3 (Levenson et al., 2004). Inhibition of ERK/MAPK signaling has also been found to impair histone acetylation regulation in an invertebrate model of food aversion learning (Danilova et al., 2010) and in the consolidation of auditory fear memories (Monsey et al., 2011). These findings implicate the interplay of traditional genomic signaling cascades and epigenetic mechanisms. They also suggest the existence of a level of epigenetic regulation of transcriptional processes necessary for memory formation, which has only become more widely appreciated within the last decade.

## Epigenetic Underpinnings of Fear Memory

### Histone acetylation mediates auditory fear memory consolidation

**Figure 2:**
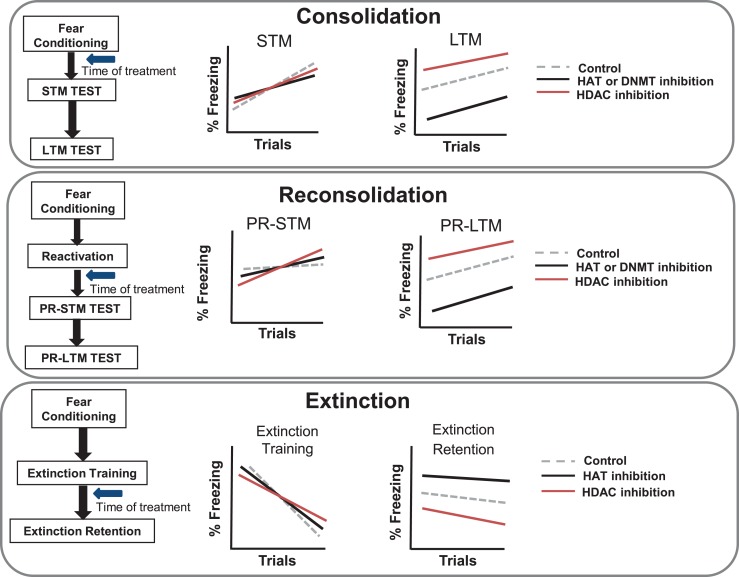
**Schematic diagram of different phases of fear learning, and predicted effects of inhibiting histone acetyltransferase, histone deacetylase, or DNA methyltransferase**. The schematic diagram illustrates the predicted outcome of pharmacological inhibition of histone and DNA modifying enzymes on the primary aspects of fear memory. (Top) When treated with inhibitors during the consolidation phase of fear conditioning, short-term memory (STM) tests are not generally effected, but long-term memory (LTM) expression is impaired with HAT and DNMT inhibition or increased with HDAC inhibition. (Middle) When inhibitors are given after a brief memory reactivation, they may affect memory reconsolidation processes. In this case, there are no predicted effects on short-term memory post-reactivation (PR-STM); however, PR-LTM is impaired with HAT and DNMT inhibition or increased with HDAC inhibition. (Bottom) When inhibitors are given following extinction training, HAT inhibition is predicted to impair extinction retention while HDAC inhibition is predicted to enhance extinction retention.

Early work examining the necessity of epigenetic mechanisms in the regulation of memory formation has largely employed hippocampal-dependent memory paradigms (Levenson et al., 2004; Miller and Sweatt, 2007; Lubin et al., 2008; Miller et al., 2008; Stefanko et al., 2009). More recent work has begun to examine the role of epigenetic mechanisms in amygdala-dependent memory processes. In agreement with the evidence for regulation of histone acetylation with contextual fear conditioning, a recent series of studies has demonstrated that auditory fear conditioning also results in an increase in histone H3 but not H4 acetylation in the LA (Monsey et al., 2011). Further, this study demonstrated that increasing histone acetylation via HDAC inhibition in the LA resulted in enhanced auditory fear memory consolidation, i.e., freezing during long-term memory was enhanced whereas short-term memory was not affected (Figure 2).

Despite the documented increase in histone H3 acetylation with auditory fear conditioning and facilitation of fear memory consolidation via HDAC inhibition, these findings did not directly address the necessity of histone acetylation in fear memory consolidation. To date, while many proteins have been identified which possess HAT activity, three in particular have been widely examined within the field of learning and memory: E1a-associated protein (p300), CREB binding protein (CBP), and p300-CBP-associated protein (PCAF) (Barrett and Wood, 2008). Much of the work examining the role of these HATs in memory processes have employed transgenic mouse models of varying degrees from brain-region specific to full knockout of HAT proteins (Oike et al., 1999; Alarcon et al., 2004; Korzus et al., 2004; Wood et al., 2005, 2006; Oliveira et al., 2007, 2011; Valor et al., 2011). From this wide collection of studies only two have revealed deficits in amygdala-dependent auditory fear memory (Oike et al., 1999; Alarcon et al., 2004), while many report deficits in the hippocampal-dependent tasks of contextual fear and the novel object recognition paradigm (Korzus et al., 2004; Wood et al., 2005, 2006; Oliveira et al., 2007, 2011; Valor et al., 2011).

Recently, pharmacological inhibitors of HAT activity have become commercially available and these agents have been employed in specific brain regions to examine more targeted inhibition of HAT proteins in conjunction with learning and memory paradigms, independent of the potential developmental confounds with transgenic models (Marek et al., 2011; Wei et al., 2012; Zhao et al., 2012). Inhibition of HAT activity using either garcinol or c646 administration accompanying auditory fear conditioning has been found to inhibit training-related changes in histone acetylation within the LA and coordinately inhibit auditory fear memory consolidation, in a temporally graded manner (Maddox et al., 2013a,b). Further, these studies demonstrated that inhibition of HAT activity shortly following auditory fear conditioning also impaired learning-related enhancements in auditory-evoked field potentials (AEFPs) within the LA, a commonly studied neurophysiological correlate of auditory fear memory consolidation (Quirk et al., 1995; Rogan et al., 1997). It is worth considering that given the large sequence homology between CBP and p300, and given the ability of CBP/p300 to structurally associate with PCAF to regulate histone acetylation (Schiltz et al., 1999), making claims about the necessity of one HAT over another using these pharmacological inhibitors is not warranted. While these data do reveal a critical role for HAT activity in the consolidation of auditory fear memories, more attention is needed to examine the specific contributions each of these HATs may make either independently or in combination with one another to promote learning and memory.

A recent study examining the role of PCAF in auditory fear memory extinction suggests that PCAF in the infra-limbic prefrontal cortex (ILPFC) has a selective role in the consolidation of auditory fear memory extinction (Wei et al., 2012). However it is worth noting that in an experiment from this study, the PCAF inhibitor H3-CoA-20-Tat was infused into the prelimbic PFC (PLPFC) immediately following auditory fear conditioning and was found to enhance consolidation of auditory fear memory. A role for the PLPFC in expression and consolidation of fear memories has been established (Vidal-Gonzalez et al., 2006; Corcoran and Quirk, 2007; Choi et al., 2010). Further, a role for brain-derived neurotrophin factor (BDNF) in the PLPFC has been identified as a key mediator of fear memory consolidation (Choi et al., 2010), and as recent work has shown that HDAC administration can enhance levels of BDNF mRNA (Zeng et al., 2011) (*and see below)*, the mechanism though which inhibition of HAT activity in the PLPFC results in enhanced fear memory consolidation remains unclear.

### DNA methylation mediates auditory fear memory consolidation

While traditionally considered a stable and enduring transcriptional constraint, studies examining the role of DNA methylation in learning and memory have revealed dynamic regulation of DNA methylation (Miller and Sweatt, 2007; Lubin et al., 2008; Feng et al., 2010; Han et al., 2010). As DNMTs serve to add methyl groups to CpG islands on DNA sequences to silence gene expression, traditional logic suggests that their inhibition would enhance memory formation via transcriptional activation. Conversely, studies have shown that inhibition of DNMT activity interferes with memory consolidation processes in a variety of hippocampal-dependent learning paradigms, including contextual fear, cocaine-induced place preference, and Morris water maze learning (Miller and Sweatt, 2007; Lubin et al., 2008; Feng et al., 2010; Han et al., 2010). In agreement with these findings, auditory fear conditioning has recently been associated with an increase in DNMT3a protein within the LA (Monsey et al., 2011). Inhibition of DNMT activity within the LA at or near the time of training results in impaired fear memory consolidation, suggesting that DNMT activity is critical for amygdala-dependent fear memory consolidation as it is in the hippocampal contextual fear memory system (Monsey et al., 2011). In support of these findings, experiments employing slice electrophysiology, revealed that inhibition of DNMT activity impairs long-term potentiation (LTP), a well established model of synaptic plasticity, from both thalamic and cortical input pathways to the amygdala. Thus, this pattern of findings clearly implicates DNMT activity in the establishment of synaptic plasticity and ultimately auditory fear memory consolidation.

While these early studies implicate a role for DNMT activity in memory consolidation processes, only recently has evidence emerged to suggest the existence of a DNA demethylase within the CNS. Gadd45b has been proposed to be a regulator of active DNA demethylation within the adult CNS (Leach et al., 2012; Sultan et al., 2012). Both of these studies employ a genetic knockout model of Gadd45b yet draw contrasting conclusions regarding the role of Gadd45b in memory processes. While one study revealed enhancements in contextual fear memory consolidation with mild and moderate training parameters and implicates a role of Gadd45b as a negative regulator of contextual fear memory formation (Sultan et al., 2012), the other study demonstrated impaired contextual fear memory consolidation and is in line with a permissive role for Gadd45b in memory formation (Leach et al., 2012). However, both studies failed to demonstrate an effect on the consolidation of amygdala-dependent auditory fear memory, suggesting that the identification of an active DNA demethylase in the auditory (or cued) fear memory system has yet to be determined. While the findings of enhanced memory formation accompanying Gadd45b knockout are in line with the findings demonstrating memory consolidation deficits associated with DNMT inhibition (Miller and Sweatt, 2007; Miller et al., 2008), there remains a lack of cohesion regarding the role of Gadd45b in contextual fear memory formation. These findings demonstrate a clear need for further investigation into the underlying mechanism through which Gadd45b may function as a regulator of memory formation.

Although multiple labs have now documented fear memory consolidation deficits accompanying DNMT activity inhibition in either the amygdala or the hippocampus, the findings are still somewhat counter-intuitive. However, a few studies have provided evidence for a possible mechanism underlying these memory deficits. A series of studies has revealed an interaction between DNA methylation and histone acetylation in the consolidation of contextual and auditory fear memories (Miller et al., 2008; Monsey et al., 2011). Both studies demonstrated that memory consolidation deficits accompanying DNMT inhibition were associated with impaired training-related regulation of histone acetylation but that consolidation and histone acetylation deficits could be rescued with HDAC inhibition. Thus, these findings suggest that one method by which DNMT inhibition may result in memory consolidation deficits is via its inhibition of the critical training-related regulation of histone acetylation and suggest that there is a critical cooperation between these epigenetic mechanisms which is necessary for memory consolidation.

Another compelling hypothesis for the memory consolidation deficits associated with DNMT inhibition concerns the differential effect on DNA-methylation patterns across various subsets of genes. First, work has suggested that the methyl-binding protein, MeCP2, interacts with the transcription factor CREB1 to regulate the activation of CRE-mediated genes, suggesting that methylation and the binding of methyl-binding proteins (MBPs) at CRE sites is associated with transcriptional activation (Chahrour et al., 2008). In agreement with this finding, work from Farah Lubin’s group (Gupta et al., 2010) demonstrated that contextual fear conditioning results in an increase in DNA methylation within the *zif268* promoter region and a corresponding increase in zif268 mRNA expression. Moreover this group demonstrated an increase in MeCP2 within the *zif268* promotor region associated with DNA methylation, suggesting that indeed methylation is required for the activation of CRE-mediated genes such as *zif268*. Thus these findings may suggest that inhibition of DNMT activity near the time of fear conditioning results in consolidation deficits via the inhibition of CRE-mediated genes which are critical for consolidation, such as *zif268* (Maddox et al., 2011), and suggest that DNA methylation can in some cases be associated with transcriptional activation.

An extension of this hypothesis concerns the possibility that DNMT inhibition may offset the balance of memory-promoting (e.g., the CRE-mediated IEG *zif268*) and memory-suppressive genes. Contextual fear conditioning has been associated with demethylation and a corresponding increase in *reelin* mRNA, a memory-promoting gene, in the hippocampus. Conversely, contextual fear learning leads to a hypermethylation of the memory-suppressive gene protein phosphatase (*PP1)* with a corresponding reduction in *PP1* mRNA (Miller and Sweatt, 2007). Further, DNMT inhibition was found to reverse these changes, such that the training-induced methylation of *PP1* was impaired and thus *PP1* mRNA was increased. These data suggest that one manner in which DNMT inhibition results in impaired memory consolidation is via the demethylation of memory-suppressing genes, such that their enhanced expression results in memory impairment. Further, it remains possible that the findings of DNMT inhibition’s effects on (1) the reduction of training-related changes in histone acetylation, (2) ability to enhance the transcription of memory-suppressive genes, and (3) its impairment of memory-promoting genes, especially the induction of CRE-mediated IEGs, are not mutually exclusive events. This suggests that these effects should be considered in concert when further examining the mechanism through which DNMT inhibition impairs memory consolidation.

## Epigenetic regulation of auditory fear memory reconsolidation

Another growing field of study within the realm of epigenetic-mediation of fear memories is the examination of a role for epigenetic processes in the reconsolidation of auditory fear memories. Reconsolidation is the phenomenon whereby retrieval of a previously acquired memory results in the induction of a period of instability during which the memory may be updated, either strengthened or weakened, prior to being re-stabilized (Nader et al., 2000; Tronson and Taylor, 2007). An early study noted the existence of epigenetic mechanisms in contextual fear memory reconsolidation by revealing the retrieval-induced regulation of histone acetylation in area CA1 of the hippocampus via the NF-κB/IKK (Nuclear Factor Kappa-light-chain-enhancer of activated B cells/inhibitor of NF-κB kinase) pathway (Lubin and Sweatt, 2007). Within the last few years, a series of studies has further contributed to this early work by outlining a critical role for epigenetic mechanisms in auditory fear memory reconsolidation. As with initial auditory fear memory consolidation, retrieval of a previously acquired auditory fear memory was found to result in a retrieval-dependent increase in histone H3 acetylation, but not regulation of H4 acetylation in the LA (Maddox and Schafe, 2011). Moreover, HDAC inhibition accompanying auditory fear memory retrieval was found to enhance memory reconsolidation in a retrieval-dependent and temporally graded manner, suggesting that as with auditory fear memory consolidation, HDAC activity appears to negatively regulate fear memory reconsolidation within the LA (Figure 2). To further explore the role of histone acetylation in fear memory reconsolidation, more recent work has revealed that HAT activity is critical in mediating retrieval-related alterations in histone acetylation and that HAT inhibition impairs fear memory reconsolidation (Maddox et al., 2013a,b). Results from both studies have demonstrated that inhibition of HAT activity results in a long-lasting and robust reconsolidation deficit which is dependent on memory retrieval, insensitive to spontaneous recovery, reinstatement, and fear renewal in a novel context. Further, these studies demonstrated that inhibition of HAT activity accompanying fear memory retrieval was capable of reversing the underlying memory-associated changes in AEFPs, suggesting that this memory intervention strategy is effective at impairing fear memory reconsolidation at the level of behavior and at the level of synaptic plasticity, *in vivo*. Moreover, as garcinol is a naturally occurring HAT inhibitor, these data were the first to document the pre-clinical efficacy of a naturally occurring pharmacological strategy in conjunction with reconsolidation-based fear memory intervention. These findings highlight the potential for future identification of naturally occurring compounds to use in the treatment of fear and anxiety disorders (Maddox et al., 2013a).

Comparatively fewer studies have examined the role of DNA methylation in auditory fear memory reconsolidation processes; however in agreement with the findings of studies of auditory fear memory consolidation, intra-LA inhibition of DNMT activity at or near the time of auditory fear memory retrieval has been shown to impair the reconsolidation of memory (Maddox and Schafe, 2011). Moreover, inhibition of DNMT activity accompanying fear memory retrieval was found to result in a retrieval-dependent, temporally graded deficit which is insensitive to spontaneous recovery, reinstatement, and fear renewal. While these data demonstrate a critical role for DNMT activity in reconsolidation as one method to alleviate existing fear memories, a role for DNA methylation and DNMT activity has yet to be revealed for the consolidation of fear extinction memory, the other widely explored method for alleviating existing fear memories.

## Epigenetic regulation of fear memory extinction

Rodent models of fear memory extinction have proven quite useful in making suggestions about relevant pharmacological manipulations which may be implemented in cognitive-behavioral therapy for the treatment of phobias, anxiety disorders, and PTSD (Andero and Ressler, 2012). Extinction involves the repeated presentation of the fear-invoking conditioned stimulus (CS) in the absence of the unconditioned stimulus (US), and results in the formation a new memory which inhibits the expression of the existing fear memory (Myers and Davis, 2007). Given that HDAC inhibitors have been widely reported to enhance memory, recent work has turned toward examining the potential for HDAC inhibition to enhance extinction memory as it may have clinical implications. A few labs have now provided evidence for the benefit of HDAC inhibition on fear extinction learning by demonstrating that systemic HDAC inhibition is capable of facilitating extinction for auditory fear memory (Lattal et al., 2007; Bredy and Barad, 2008; Fujita et al., 2012; Itzhak et al., 2012) (Figure 2) and facilitating extinction of cocaine conditioned place preference (Malvaez et al., 2010).

However, there is some evidence to suggest that there may be limits on the effectiveness of HDAC inhibition in facilitating extinction. One such study demonstrated that intra-hippocampal or intra-ILPFC administration of the HDAC inhibitor sodium butyrate (NaB) was capable of enhancing contextual fear memory extinction, but only under conditions when extinction training was weak, i.e., NaB administration was not effective in facilitating extinction using more stringent extinction sessions (Stafford et al., 2012). The lack of a facilitation of extinction with HDAC inhibition in conjunction with stronger extinction parameters may suggest the presence of a ceiling effect. However, extinction facilitation accompanying HDAC inhibition has not always proven successful (Kilgore et al., 2010). Another study has suggested that while overexpression of HDAC1 in the hippocampus facilitates contextual fear extinction, inhibition of HDAC1 correspondingly results in impaired extinction (Bahari-Javan et al., 2012). As HDAC inhibition has been associated with enhanced extinction in a number of studies using pharmacological approaches such as NaB, Vorinostat, or TSA, HDAC inhibitors which inhibit multiple classes of HDACs, it remains unclear if different classes of HDACs make different contributions to fear memory extinction. The lack of a cohesive role for HDAC activity in fear memory extinction clearly suggests that this is an area of work which requires further investigation.

Another study has demonstrated that inhibition of PCAF activity within the ILPFC impairs the consolidation of extinction for an auditory fear (Wei et al., 2012), data which implicate a critical role for histone acetylation via HATs in fear memory extinction. However, it is worth noting that while many studies have demonstrated extinction enhancements accompanying HDAC inhibition, one study has shown that inhibition of the HAT activity of p300/CBP paradoxically enhances extinction memory (Marek et al., 2011). The existence of the same behavioral outcome (i.e., enhanced extinction via both inhibition and enhancement of histone acetylation) is somewhat perplexing and further research is warranted to uncover the mechanism underlying these findings. As this lab has provided evidence for a critical role for PCAF in the consolidation of auditory fear extinction (Wei et al., 2012) further investigation might reveal a unique relationship between these HATs and their role in the regulation fear memory extinction.

Intriguingly, a recent study examining the potential chromatin modifications in the ventral-medial PFC (vmPFC) occurring with extinction of conditioned place aversion (CPA) accompanying morphine withdrawal (Wang et al., 2012) demonstrated increased AcH3 and AcH4 following extinction. This study further demonstrated that intra-vmPFC administration of the partial NMDA-receptor agonist d-cycloserine (DCS) resulted not only in enhanced CPA extinction, but also facilitated extinction-related increases in AcH3 and AcH4. As there is already evidence for the efficacy of DCS in the treatment of fear memory in rodent models (Walker et al., 2002) and in phobias in a clinical population (Ressler et al., 2004), these data may suggest that one unappreciated mechanism through which DCS may be effective in promoting extinction-related plasticity is via enhancement of chromatin modifications which are downstream of NMDAR activation.

## Translating Animal Models to the Clinic: Epigenetic Modifications of Genes Associated with Fear and Anxiety Disorders

Much work has revealed the regulation of global epigenetic alterations, especially histone acetylation and DNA methylation, accompanying both auditory fear conditioning and memory retrieval in the amygdala and accompanying contextual fear conditioning in the hippocampus. Additionally, some progress has been made in revealing the epigenetic regulation of specific genes associated with fear memory. Animal models examining the impact of early-life trauma, stress, and adversity as a risk factor for the future development of psychiatric disorders appear to have gained support from recent studies in human clinical populations. Moreover, it is the examination of epigenetic-modifications associated with specific genes which has recently been explored within the context of clinical PTSD and other psychiatric disorders. Given recent work examining epigenetic risk factors associated with the development of PTSD, we now review work employing animal models of epigenetic regulation of anxiety and fear associated genes which has been translated into human clinical populations, including epigenetic modifications of glucocorticoid receptor (GR) function including *FKBP5*, *Nr3c1*, and the *BDNF* pathways.

## The BDNF-TrkB Pathway and Its Downstream Effectors

Brain-derived neurotrophin factor is a neurotrophic factor which has been widely implicated in nervous system development, synaptic plasticity, and has been shown to be highly enriched in brain-regions associated with emotional learning including the amygdala, hippocampus, and PFC (Hofer et al., 1990). Importantly, support for a role for BDNF in emotional learning in animal models has been recently translated to PTSD in humans, as illuminated by the finding that individuals with the BDNF polymorphism Val66Met have impaired fear memory extinction (Soliman et al., 2010). While a role for the BDNF peptide and its receptor, TrkB, have been well established in auditory fear conditioning (Rattiner et al., 2004; Choi et al., 2010; Andero et al., 2011), additional insight into the epigenetic mechanisms regulating the BDNF pathway and its role in PTSD have been investigated by a number of groups.

Brain-derived neurotrophin factor consists of nine 5′-non-coding exons, each possessing individual promoter regions and one 3′-coding exon (IX) which codes for the BDNF pre-protein amino acid sequence (Aid et al., 2007). The existence of these nine individual promoter regions within the BDNF gene sequence allows for the potential for unique BDNF transcripts with differential regulatory regions and has been proposed to ultimately underlie functional differences in BDNF transcription (e.g., Rattiner et al., 2004). Due to this potential for functional differences in BDNF accompanying differential regulation of individual BDNF exons, studies exploring the epigenetic regulation of BDNF have examined multiple BDNF exons. Using a contextual fear memory paradigm, it was determined that context-shock associations resulted in a selective decrease in DNA methylation within BDNF exon IV and a corresponding increase in BDNF exon IV mRNA in the hippocampus (Lubin et al., 2008). In addition, a study employing a PTSD model, examining the impact of prior-stress experience on subsequent contextual fear memory formation, determined that prior-stress experience resulted in enhanced freezing levels and correspondingly an increase in BDNF exon I and IV mRNA in the hippocampus (Takei et al., 2011). This study further revealed an increase in histone H3 and H4 acetylation within exon I and IV BDNF promoters, implicating a role for histone acetylation-mediated enhancement of BDNF mRNA accompanying stress-enhanced contextual fear memory formation. While this study is in agreement with the previous implication of epigenetic regulation of BDNF exon IV in contextual fear memory formation, the finding of significant regulation of BDNF exon I regulation with stress-enhanced contextual fear learning may suggest that stress allows for more extensive epigenetic modifications within the BDNF gene to promote fear memory formation. However, it is worth noting that a previous study from this group examining the epigenetic regulation of BDNF following a single session of immobilization stress in the hippocampus revealed a reduction in AcH3 association with BDNF exon I and IV promoters and a corresponding decrease in BDNF I and IV transcript mRNA 2 h following stress, but not 24 h (Fuchikami et al., 2009). As these findings suggest that previous stress can enhance histone acetylation-mediated BDNF transcription accompanying contextual fear conditioning 1-week later (Takei et al., 2011), the immediate post-stress reduction in BDNF transcription, and histone-modification seems at odds with this hypothesis. Thus, more work is necessary to examine how prior-stress experience can enhance histone acetylation-mediated BDNF transcription at this later time point, when stress experience itself does not appear to have a long-lasting (24 h later) effect on BDNF transcription in the hippocampus.

Although studies examining the regulation of histone acetylation-mediation of BDNF transcription have not revealed enduring changes in acetylation-mediated regulation of specific BDNF promoter transcripts, suggesting that chromatin modifications are transiently mediated following stress and/or fear conditioning, work examining DNA-methylation of BDNF promoters has revealed more enduring epigenetic regulation of BDNF. Using a psychosocial-stress PTSD model in rats, methylation of BDNF promoters was examined following a 1 month stress period (Roth et al., 2011). BDNF exon IV was found to have enhanced levels of CpG methylation in both the dentate gyrus and area CA1 of the hippocampus in the stress exposed group and a corresponding reduction in BDNF exon IV mRNA levels. However, this study failed to find stress-related alterations in BDNF DNA methylation in the amygdala and PFC. These data suggest that there is a robust regulation of DNA methylation at CpG islands within BDNF exon IV in the hippocampus which is a consequence of stress. It remains unclear why no changes were observed in the amygdala and PFC, both structures which have been shown to be affected by experience with stress (Rodrigues et al., 2009). However, the epigenetic-regulation of BDNF exon IV in the hippocampus is in agreement with the aforementioned findings and signifies that exon IV in particular undergoes robust epigenetic-modifications accompanying contextual fear memory formation and stress exposure. Thus this BDNF promoter/exon regulation is likely key in the development of a PTSD-like condition in these animal models.

Translational work employing a rodent model of early-life maltreatment and trauma has demonstrated long-lasting epigenetic-mediation of BDNF transcription (Roth et al., 2009). In this study, infant rats were exposed to a stressed, “abusive” mother for 30 min per day during the first post-natal week or were cross-fostered by a non-abusive mother for the same period of time. Upon adulthood it was determined that those rats who experienced maltreatment by the abusive mother had significant reductions in total BDNF mRNA (exon IX) in the hippocampus, compared to the cross-fostered non-maltreated adults, suggesting that there is a long-lasting effect of early-life maltreatment on BDNF regulation. Further, this study revealed an increase in BDNF exon IV and IX DNA methylation in the PFC of adult rats with a history of early post-natal maltreatment, suggesting that early-life stress is capable of resulting in long-lasting epigenetic changes at BDNF promoters which result in long-lasting BDNF transcription deficits.

These studies reveal a role for epigenetic-mediated regulation of BDNF transcription accompanying contextual fear memory formation, and demonstrate an enhancement of epigenetic-mediated BDNF transcription with prior-stress experience. To date only one study has examined the potential role for epigenetic-mediation of BDNF transcription accompanying fear memory extinction (Bredy et al., 2007). This study demonstrated that while auditory fear conditioning was associated with increased AcH3 at BDNF I and IV exon promoters in the PFC, extinction was associated with a decrease in AcH3 recruitment to the exon I promoter but increased AcH4 at BDNF exon IV promoter. Further, while exon IV mRNA was enhanced with fear conditioning, extinction was found to result in a more profound increase in exon IV mRNA and also to induce an increase in exon I mRNA. These data are the first to reveal differential regulation of BDNF transcripts in the PFC accompanying fear memory formation and extinction learning and suggest that there may be different epigenetic-mediation of BDNF transcription for different forms of memory, i.e., consolidation versus extinction.

**Figure 3:**
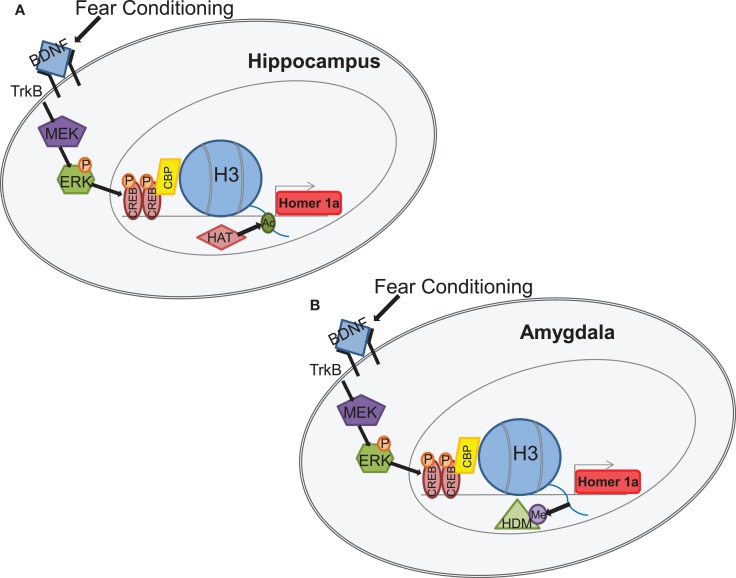
**Example of BDNF-mediated transcriptional activation, through epigenetic regulation of the homer1a synaptic plasticity gene in hippocampus and amygdala (Mahan et al., 2012)**. Fear conditioning rapidly increases BDNF-signaling in both the hippocampus and amygdala and results in activation of the TRKB pathway that activates MEK and ERK. ERK further phosphorylates CREB which is then translocated to the nucleus where it binds to CRE sites in the promoter region of *homer1a*. CREB subsequently recruits CBP which induces specific histone changes **(A)** increased acetylation in the hippocampus and **(B)** decreased methylation in the amygdala, both of which result in increased *homer1a* transcription (Figure courtesy of Amy Mahan, PhD).

While these early studies have laid a firm foundational role for epigenetic regulation of BDNF accompanying fear memory and/or stress exposure in a variety of paradigms, recent work has identified epigenetic regulation of *homer1a*, a gene variant of *homer1* and a downstream transcriptional target of BDNF-TrkB signaling (Mahan et al., 2012). Using a fear conditioning paradigm which is capable of instating both auditory and contextual fear memory-related plasticity, this study revealed that fear conditioning was associated with an increase in histone H3 acetylation within the *homer1* promoter region within the hippocampus while a decrease in histone H3 methylation within the *homer1* promoter region was observed within the amygdala (Figure 3). Both of these changes in histone H3 modifications were associated with an increase in *homer1a* mRNA, suggesting that there is differential and brain-region specific epigenetic regulation of *homer1a* expression. As with the earlier suggestion of differential epigenetic regulation of BDNF transcription for fear memory formation and extinction memory in the PFC (Bredy et al., 2007) these data may suggest that contextual fear memories mediated by the hippocampus may require histone acetylation-mediated *homer1a* transcription, whereas auditory fear memories mediated by the amygdala may require a decrease in H3 methylation to induce critical regulation of *homer1a* with memory formation.

As noted earlier, while a variety of animal models of fear and anxiety disorders have demonstrated epigenetic regulation of BDNF, only a few studies have revealed the potential role for epigenetic regulation of BDNF in human clinical populations. For example, a pair of studies has examined post-mortem differences in epigenetic regulation of BDNF in suicidal behavior (Keller et al., 2010, 2011). Examination of BDNF in Wernike’s area revealed increased BDNF promoter IX methylation with a corresponding decrease in BDNF mRNA and protein levels in suicide subjects (Keller et al., 2010). Despite this finding, no relationship with TrkB promoter methylation and suicide was revealed (Keller et al., 2011). The failure to find epigenetic regulation of TrkB receptor in suicidal behavior may be supported by the lack of consistent findings regarding regulation of TrkB expression in stress-mediated rodent models of PTSD (Smith et al., 1995a,b; Takei et al., 2011). Examination of post-mortem epigenetic modifications in depressed patients who underwent antidepressant treatment has also been associated with a decrease in H3K27 trimethylation at BDNF promoter IV in the PFC and subsequent increased BDNF mRNA. However, no difference in H3K27 was observed within BDNF promoter IV for those patients not on antidepressant treatment (Chen et al., 2011). While these studies suggest a correlation between post-mortem epigenetic modifications at BDNF promoters, it is unclear how these differences relate to functional differences in BDNF transcription which underlie depression or suicide behavior *in vivo*.

Despite the suggestion of a role for epigenetic modulation of BDNF activity in depression and suicide, only one study has demonstrated a modest association between increased methylation of one BDNF CpG site with current PTSD status (Smith et al., 2011). Moreover, although previous reports have noted lower levels of BDNF from blood serum in PTSD populations (Dell’Osso et al., 2009; Berger et al., 2010; but see Hauck et al., 2009), it is unclear how this reduction relates to either a risk factor for the development of PTSD or is rather a consequence of PTSD. Despite this preliminary evidence, a recent model relating genetic risk factors associated with impaired fear extinction such as the BDNF val66met polymorphism, potential epigenetic-modifications associated with early-childhood adversity and trauma, and adulthood trauma has emerged to implicate a role for BDNF function in the comorbidity of PTSD and bipolar disorder (Rakofsky et al., 2012). This model, with support from animal studies suggests that there may be a “critical period” in development during which early-life trauma or stress can most readily result in epigenetic modifications on genes associated with stress and PTSD which later impact the likelihood for the future development of PTSD. In support of this model, data have demonstrated epigenetic-modifications associated with genes which regulate GR function in early-childhood adversity and subsequent risk for the development of PTSD. Further, as work has suggested that glucocorticoid exposure can influence BDNF expression (Gourley et al., 2009), it is likely that with further examination of these genes and the interactions amongst them, a role for epigenetic modifications of BDNF may be revealed for PTSD.

## Epigenetic Modifications of FKBP5 and Glucocorticoid Receptor Function

**Figure 4:**
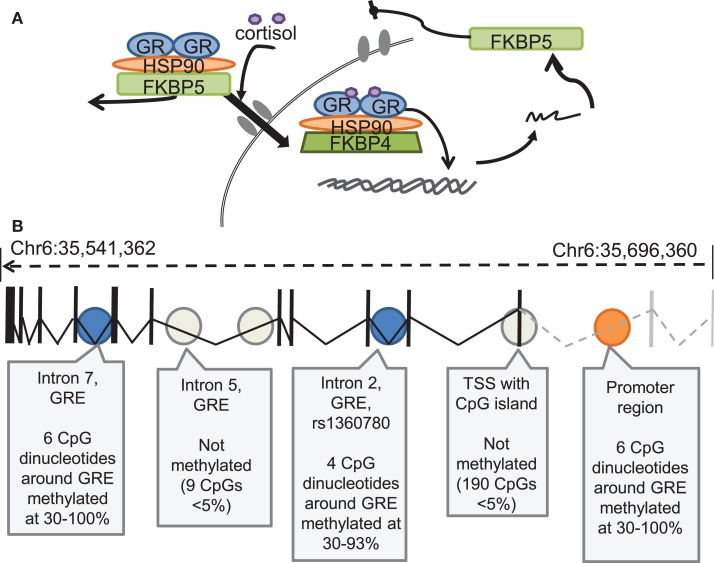
**FKBP5 regulation of GR function, and DNA methylation regulatory sites within the *FKBP5* Gene (A) Schematic diagram of FKBP5 regulation of Glucocorticoid Receptor (GR) function**. FKBP5 protein acts as an inhibitory chaperone, preventing GR translocation to the nucleus. With increasing cortisol binding, FKBP5 is displaced by FKBP4, allowing for translocation and gene activation, with more FKBP5 mRNA produced as one of the GR-sensitive genes, completing the intracellular negative feedback loop. *(Figure courtesy of Elisabeth Binder)*. **(B)** DNA methylation of the FKBP locus, in which significant DNA methylation was observed in the promoter region, Intron 2 and intron 7 of the FKBP5 gene (Figure adapted from Klengel et al., 2013).

A role for the hypothalamic-pituitary adrenal (HPA) axis in stress and fear memory has been well appreciated (Roozendaal et al., 2009), and one important target of HPA activity is the regulation of GR activity. GR translocation is mediated by the GR co-chaperone protein FK506 binding protein 5 (*FKBP5*) which is associated with the chaperone heat shock protein 90 (hsp90) to form a chaperone complex which regulates GR dynamics (Hubler and Scammell, 2004). Typically *FKBP5* is regulated via a negative feedback loop such that GR activation promotes an increase in *FKBP5*, which limits GR translocation to the nucleus and GR-dependent transcriptional activation (Binder, 2009; Figure 4). Many *FKBP5* polymorphisms have been identified and evidence has emerged to suggest that these polymorphisms impact cortisol response. Such findings suggest that individuals carrying these polymorphisms may have maladaptive responses to stress and thus may be at enhanced risk for developing psychiatric disorders (Ising et al., 2008; Mahon et al., 2010; Touma et al., 2011). Indeed HPA dysregulation and GR resistance, mediated by alterations in the *FKBP5*-governing negative feedback loop, have been discussed as a key endocrine marker for mood disorders (Binder, 2009). In agreement with this hypothesis, a recent study revealed differences in gene expression profile responses in depressed patients versus healthy controls using a dexamethasone-suppression test (Menke et al., 2013), a tool commonly used to measure GR resistance. This study revealed that depressed patients had much higher cortisol levels 21 h following dexamethasone (dex) intake compared to controls, suggesting GR resistance in depressed patients. This is in contrast to the enhanced GR-sensitivity typically seen in PTSD patients. Further, this study explored gene expression profiles from peripheral blood accompanying dex treatment. It was found that depressed patients had reduced levels of *FKBP5* following dex treatment compared to controls, a pattern of findings which suggest impaired GR sensitivity and *FKBP5* expression in depressed patients. Additionally these findings confirm the usefulness of dexamethasone-stimulated gene expression profiles as a biomarker tool to uncover depression-related alterations in GR reactivity.

Animal models of stress have also noted a role for *FKBP5* in coping behaviors and in the mediation of stress effects. Studies employing *FKBP5* knockout mice have demonstrated reduced HPA reactivity and highlighted a role for *FKBP5* in mediating coping behaviors to stress (Touma et al., 2011; Hartmann et al., 2012). Further, a study employing a chronic corticosterone exposure paradigm, previously shown to result in a phenotype similar to chronic stress exposure (Gourley et al., 2009) demonstrated an increase in FKBP5 mRNA in the hippocampus, hypothalamus, and blood of mice with a correspondent decrease in FKBP5 intron 5 DNA methylation (Lee et al., 2010). This group further determined that this chronic corticosterone exposure paradigm also resulted in alterations in FKBP5 methylation, as evident from blood samples which correlated with glucocorticoid load and with anxiety-like behaviors as measured by time spent in the closed arms of an elevated plus maze (Lee et al., 2011). Thus these findings highlight the consequences of prolonged corticosterone exposure on levels of FKBP5 and the ability to correlate epigenetic modifications of *FKBP5* in blood samples with behavioral consequences associated with a stress phenotype. Moreover, FKBP5 mRNA has been shown to be increased following stress, especially in areas already associated with stress and emotional reactivity, including the paraventricular nucleus, central nucleus of the amygdala, and hippocampus, regions which express low basal levels of FKBP5 mRNA (Scharf et al., 2011). However, it is important to note that studies in humans have revealed lower levels of *FKBP5* and GR mRNA in the amygdala of suicide victims (Perez-Ortiz et al., 2012) and lower levels of FKBP5 mRNA in the blood of PTSD patients (Yehuda et al., 2009). These data suggest that further work is needed to address the mechanisms through which shortened stress exposure as is likely in animals models leads to an increase in *FKBP5* expression, while chronic and longer lasting stress, as is likely the case in human suicide victims result in a reduction of *FKBP5*. Despite this potential contradiction, these findings further suggest that *FBKP5* is critical for stress reactivity, and that prior-stress experience may impair levels of *FKBP5* to result in poor adaptation to future stress. Additionally, as was mentioned above, human patients with depression have been consistently found to have GR non-responsiveness and those with PTSD to have GR hypersensitivity. Some of the differences in these studies may actually be due to increased *FKBP5* function associated with GR non-responsiveness, and decreased *FKBP5* associated with GR hypersensitivity. Thus, the apparent variance across studies may be due to our yet incomplete understanding of the differential biology of depression vs. PTSD.

In light of these findings, nice parallels can be seen in work examining *FKBP5* polymorphisms and the epigenetic regulation of *FKBP5* in human populations. First, an association between the *FKBP5* polymorphism rs9470080 and its interaction with early-childhood stress has been found in the risk for development of PTSD in an African American population (Binder et al., 2008; Xie et al., 2010). In support of this finding, distinct *FKBP5* genotypes have been shown to define distinct PTSD subtypes, such that while baseline cortisol levels were found to be reduced in non-risk allele carrying PTSD patients, only patients carrying the *FKBP5* polymorphism risk-allele, rs9296158, show GR hypersensitivity as measured by increased dex suppression (Mehta et al., 2011). Further evidence for a role of *FKBP5* polymorphisms in risk for PTSD development can be observed in a recent study which demonstrated that *FKBP5* genotype and methylation patterns are associated with GR sensitivity and early-childhood trauma exposure (Klengel et al., 2013). The authors found that epigenetic modifications which likely result from early-life trauma enhance *FKBP5* genotype-dependent effects on GR sensitivity and ultimately regulate risk or predisposition for the development of PTSD or depression.

In further support of a role for glucocorticoid-system mediated alterations with PTSD, additional work has revealed a role for epigenetic regulation of the gene encoding the GR *Nr3c1*, and its polymorphism *Bcl1* in fear, stress, and anxiety. A study revealed that suicide victims with a history of early-childhood abuse have differential DNA-methylation patterns around transcription start sites within the *Nr3c1* locus in the hippocampus, whereas no difference was observed between suicide-completers in the absence of early-childhood abuse or control subjects (Suderman et al., 2012). Moreover, suicide victims with a history of child abuse have been found to have reduced hippocampal GR expression and an increase in *Nr3c1* promoter methylation (McGowan et al., 2009). Additionally it has been demonstrated that increased *Nr3c1* promoter methylation levels obtained from peripheral blood samples of bipolar disorder, manic depressive disorder, and PTSD subjects correlate with the severity of childhood abuse (Perroud et al., 2011). These findings are supported by additional studies of DNA methylation of *Nr3c1* where leukocyte DNA-methylation pattern differences associated with early-childhood stress correlate with attenuated cortisol responsiveness following a Dex/CRH test (Tyrka et al., 2012).

Examination of the impact and lasting influence of early-life stress has revealed differential methylation patterns of the *Nr3c1* promoter obtained from peripheral blood samples in mothers and newborns from the Democratic Republic of the Congo (Mulligan et al., 2012). In this region plagued with war, stress-related to ongoing war was most highly correlated with low birth weight. Further it was revealed from umbilical cord blood samples that newborns from stressed mothers have increased levels of *Nr3c1* promoter methylation (Mulligan et al., 2012). These data are the first of which we are aware to show the earliest evidence of epigenetic modifications of the *Nr3c1* gene associated with early-life stress. These findings underlie the utility of these biomarker approaches to identify epigenetic-modifications associated with early-life trauma which may influence susceptibility for the future development of psychopathology including PTSD. Additional support for a role of *Nr3c1* in risk for PTSD comes from a pair of studies demonstrating that individuals with the *Nr3c1* polymorphism *Bcl1* have been shown to have enhanced emotional memory recall (Ackermann et al., 2012), have lower levels of basal plasma cortisol levels, and recall more traumatic memories from time spent in intensive-care units following cardiac surgery (Hauer et al., 2011). However, it is worth noting that a study examining GR polymorphisms in a cohort of Vietnam veterans found no association of genotype with alterations in GR responsiveness, nor was any difference in baseline cortisol level observed between PTSD and non-PTSD veterans (Bachmann et al., 2005).

While no epigenetic modifications to the *Nr3c1* gene itself have been specifically linked to PTSD diagnosis, the mounting evidence for a role of early-life stress in mediating susceptibility for the later development of PTSD, evidence for epigenetic alterations associated with suicide, and data showing early-life stress mediation of epigenetic modifications in *Nr3c1* suggest that a critical link may eventually be revealed. Further evidence for this potential link comes from another study employing a biomarkers approach which demonstrated lower levels of methylation of the *TPR* gene, which has been linked to *Nr3c1* transcription, in individuals with PTSD (Smith et al., 2011).

The human clinical data discussed so far have parallels with animal studies modeling the effects of stress on epigenetic modulation of genes as well as the impact of stress on future experience with trauma. However, there have been some very interesting findings revealing epigenetic modifications solely from human cohorts which are worth mentioning. A series of studies from the Detroit Neighborhood Health Study (DNHS) has revealed a role for epigenetic modifications of *SLC6A3* and *SLC6A4*, genes which encode for dopamine and serotonin transporters, respectively (Koenen et al., 2011; Chang et al., 2012). Individuals with a polymorphism of the *SLC6A3* gene were found to be at risk for the development of PTSD. However, individuals with methylation of this polymorphism were found to be at even greater risk for PTSD (Chang et al., 2012), suggesting that epigenetic alterations in the gene associated with the dopaminergic system may underlie risk for PTSD. Further work from this group established a potential protective role for epigenetic modifications to the *SLC6A4* gene (Koenen et al., 2011). This study determined that *SLC6A4* hypermethylation was associated with protection from the influence of multiple traumas on risk for development of PTSD, suggesting for the first time that epigenetic alterations may be protective against PTSD development. While these data suggest epigenetic-modifications associated with dopaminergic and serotonergic function in both risk and protection from PTSD development, it is important to consider the possibility that epigenetic modifications from a single cohort, such as the DNHS, may have a different epigenetic landscape than other cohorts due to the influence of social and environmental factors which can leave their mark on the epigenome. Therefore, as progress in revealing epigenetic modifications in human cohorts continues, there is a great need to examine the reliability of these modifications across multiple cohorts in order to make the most confident predictions of potential risk factors associated with PTSD development. In agreement with this notion, work from the DNHS has exposed an association between epigenetic alterations of inflammatory and immune response genes with PTSD risk (Uddin et al., 2011a,b), a pattern of findings which has been replicated in an inner-city Atlanta population (Smith et al., 2011) and suggests that an increase in methylation of anti-inflammatory genes, but decrease in methylation for pro-inflammatory genes is associated with PTSD. Thus these data suggest, across cohorts, that inflammation response genes and epigenetic-modifications associated with these genes may underlie PTSD.

## Future Directions

Great progress has been made in revealing both dynamic regulation of epigenetic modifications including changes in histone acetylation and DNA methylation, which underlie the initial formation of aversive and enduring fear memories in Pavlovian fear paradigms, as well as the enduring epigenetic-modifications associated with early-life stress. While human studies have parallels with these animal studies of early-life stress, very little (if anything at all) is known about the presence of such dynamic epigenetic regulation in humans accompanying the initial formation of traumatic memories. Additionally, while animal and post-mortem studies have enabled the ability to demonstrate epigenetic modifications in the brain accompanying fear memory formation, stress, or trauma exposure, it is unclear how these findings correlate to the recent emergence in biomarkers-based epigenetic approaches.

As highlighted throughout the work presented, some foundational progress has been made in identifying a vital role for the epigenetic mechanisms required for initial fear memory consolidation. Initial findings have also noted a role for epigenetic mechanisms in fear memory reconsolidation and while the translation of reconsolidation-based memory interventions to the clinic is still in its infancy, the emergence of a role for epigenetics in memory reconsolidation opens up an additional set of epigenetic-modifying compounds for use in the clinic in combination with reconsolidation-based therapy. As rodent models of fear extinction learning have found great translational support, these preliminary findings of epigenetic mediation of extinction also propose that epigenetic-modifying compounds may be beneficial for the treatment of fear and anxiety disorders when administered in conjunction with exposure-based psychotherapy. Finally, the translation of animal models examining epigenetic modifications related to early-life stress into studies examining the epigenetic modifications of genes associated with risk for PTSD supports continued research in the area such that therapeutic approaches targeting these epigenetic modifications may be developed not only as potential prevention options but to expand the current treatments available.

## Conflict of Interest Statement

The authors declare that the research was conducted in the absence of any commercial or financial relationships that could be construed as a potential conflict of interest.
